# TMB and TCR Are Correlated Indicators Predictive of the Efficacy of Neoadjuvant Chemotherapy in Breast Cancer

**DOI:** 10.3389/fonc.2021.740427

**Published:** 2021-12-07

**Authors:** Hongling Liang, Jia Huang, Xiang Ao, Weibang Guo, Yu Chen, Danxia Lu, Zhiyi Lv, Xiaojun Tan, Weixing He, Ming Jiang, Haoming Xia, Yongtao Zhan, Weiling Guo, Zhiqing Ye, Lei Jiao, Jie Ma, Changxi Wang, Hongsheng Li, Xuchao Zhang, Jianqing Huang

**Affiliations:** ^1^ Department of Breast Oncology, Affiliated Cancer Hospital & Institute of Guangzhou Medical University, Guangzhou, China; ^2^ School of Health Management, Guangzhou Medical University, Guangzhou, China; ^3^ Guangdong Lung Cancer Institute, Cancer Center, Guangdong Provincial People’s Hospital, Guangdong Academy of Medical Sciences, School of Medicine, South China University of Technology, Guangzhou, China; ^4^ Department of Pathology, Affiliated Cancer Hospital & Institute of Guangzhou Medical University, Guangzhou, China; ^5^ Panovue Biological Technology Co., Ltd, Beijing, China; ^6^ Geneplus-Shenzhen, Shenzhen, China

**Keywords:** breast cancer, tumor mutational burden, tumor-infiltrating lymphocytes (TILs), neoadjuvant chemotherapies (NACs), T-cell receptors (TCRs)

## Abstract

Immune characteristics were reported correlated to benefit neoadjuvant chemotherapy (NAC) in breast cancer, yet integration of comprehensive genomic alterations and T-cell receptors (TCR) to predict efficacy of NAC needs further investigation. This study simultaneously analyzed TMB (Tumor Mutation Burden), TCRs, and TILs (tumor infiltrating lymphocyte) in breast cancers receiving NAC was conducted in a prospective cohort (*n* = 22). The next-generation sequencing technology-based analysis of genomic alterations and TCR repertoire in paired breast cancer samples before and after NAC was conducted in a prospective cohort (*n* = 22). Fluorescent multiplex immunohistochemistry was used to stain CD4, CD8, PD1, TIM3, and cytokeratins simultaneously in those paired samples. TMB in pretreatment tumor tissues and TCR diversity index are higher in non-pCR patients than in pCR patients (10.6 vs. 2.3; *p* = 0.043) (2.066 vs. 0.467; *p* = 0.010). TMB and TCR diversity index had linear correlation (*y* = 5.587*x* − 0.881; *r* = 0.522, *p* = 0.012). Moreover, infiltrating T cells are significantly at higher presence in pCR versus non-pCR patients. Dynamically, the TMB reduced significantly after therapy in non-pCR patients (*p* = 0.010) but without TCR index change. The CDR3 peptide AWRSAGNYNEQF is the most highly expressed in pre-NAC samples of pCR patients and in post-NAC samples of non-pCR patients. In addition to pCR, high clonality of TCR and high level of CD8^+^ expression are associated with disease-free survival (DFS). TCR index and TMB have significant interaction and may guide neo-adjuvant treatment in operable breast cancers. Response to NAC in tumors with high TCR clonality may be attributable to high infiltration and expansion of tumor-specific CD8 positive effector cells.

## Introduction

The incidence and mortality of breast cancer (BC) remain high (1–3). Neoadjuvant therapies are standard of care for early operable diseases. Complete tumor regression and pathological complete response (pCR) have been associated with improved survival (4–6). However, improving the molecular prediction of pCR by integration of different biomarkers in a clinical neoadjuvant setting remains challenging.

In addition to the clinical genotyping of hormone receptors (ER, PR) or HER2 (7), other biomarkers of genomic alterations (8, 9) and tumor-infiltrating lymphocytes (TILs) (10–12) were reported in association with pCR of neoadjuvant therapies. Over the past years, TIL levels had also been identified as an independent predictor of pCR in genotypes of BC (13, 14). These data need further validation to be translated into clinical use.

During cancer development and evolution, the tumor microenvironment (TME) is extremely complex and dynamic, reshaped by different types of immune cells, stromal cells, and tumor cells and the interplay among them. Molecular characteristics of cancer immune suppressive microenvironment are being extensively investigated for prediction or prognosis. Three phenotypes of immune microenvironment have been proposed as inflamed, immune excluded, and immune desert. Studies on the molecular mechanisms underlying the phenotypes may provide critical biomarkers or targets for immune treatments. Presently, major immune biomarkers include tumor mutational burden (TMB), TCR diversity, HLA expression, and IFNγ signatures. Simultaneous detection of TMB, TCR, and immune cells may generate good evidence for explanation of TME. These biomarkers were shown useful and predictive to immune therapies in advanced cancer diseases, yet need further simultaneous and integrative investigation in early-stage disease. Interaction of different markers like TCR and mutation burden remains to be understood.

In this study, we studied the TMB, TCR, and counting of immune cells in the TME and correlated with clinical outcome of breast cancer patients treated with neoadjuvant therapies.

## Patients and Methods

### Patient Selection and Tumor Specimens

A prospective cohort of 22 cases were included from June 2017 to December 2018. Inclusion criteria were stage IIB to IIIC BC patients, receiving neoadjuvant chemotherapy (NAC) with informed consent of biomarker testing (**Table 1**). All those BC patients preoperatively treated with the TEC or EC followed by T regimen (T: docetaxel/liposome paclitaxel, E: pirarubicin/epirubicin, C: cyclophosphamide) for 2–8 cycles. Only two HER2-positive patients received additional treatment with trastuzumab or pertuzumab.

**Table 1:** Clinical characteristics of 22 breast cancer patients with paired samples.

ID	Age	pCR	MPR	Clin ORR	Menopause	ER	PR	Her-2	Ki-67	Disease Stage	Mole Type	His Grade	NAC	NAC Cycle	CD3^+^	CD8^+^	CD8^+^PD1^+^
**P1**	52	Yes	Yes	CR	No	+	+++	–	15%	IIIA	2	2	EC-T	4	0.172	0.014	0.0001
**P2**	48	No	Yes	PR	No	–	++	–	30%	IIIA	2	2	EDEC	8	0.059	0.001	0.000
**P3**	44	No	No	SD	No	++	++	+	30%	IIIA	2	2	DEC	8	0.105	0.011	0.0001
**P4**	58	No	No	PR	Yes	+++	+++	+++	30%	IIIA	2	2	TEC	8	0.324	0.023	0.000
**P5**	40	No	Yes	PR	No	–	–	+++	70%	IIIC	3	2	EC-T	8	0.051	0.001	0.000
**P6**	23	No	No	PR	No	–	–	–	20%	IIIA	4	2	DAC	6	0.045	0.003	0.0003
**P7**	57	No	Yes	PR	Yes	–	–	+++	40%	IIIC	3	2	TEC	6	0.085	0.009	0.0001
**P8**	48	Yes	Yes	CR	No	++	+++	+++	20%	IIIA	2	2	DAC	6	0.177	0.029	0.0004
**P9**	50	No	Yes	PR	No	–	–	+++	40%	IIIA	3	2	DAC	8	0.024	0.000	0.000
**P10**	69	No	No	PR	Yes	–	–	+++	30%	IIIC	3	2	DEC	4	0.031	0.0002	0.000
**P11**	30	Yes	Yes	CR	No	+++	++	–	70%	IIIA	2	2	TEC	7	0.081	0.033	0.002
**P12**	62	No	No	PR	Yes	+++	–	+	5%	IIIC	1	2	TEC	6	0.007	0.003	0.0001
**P13**	50	No	No	PR	No	+	–	+++	35%	IIB	2	2	TEC	3	0.042	0.059	0.019
**P14**	42	No	No	SD	No	–	–	++	5%	IIIA	3	3	TEC	6	0.019	0.001	0.00001
**P15**	28	No	No	PR	No	++	++	–	20%	IIIA	2	3	DEC	5	0.192	0.002	0.0003
**P16**	64	No	No	PR	Yes	–	+	+++	20%	IIIC	2	3	DEC	6	0.023	0.014	0.0001
**P17**	41	No	No	PR	No	+++	++	+	35%	IIIC	2	2	TEC	8	0.006	0.002	0.0001
**P18**	58	No	No	PD	Yes	–	–	+++	30%	IIIC	3	2	HT-EC	4	0.024	0.000	0.000
**P19**	52	No	No	PD	Yes	–	–	+++	10%	IIB	3	2	TH-EC	6	0.029	0.001	0.00001
**P20**	46	No	No	PR	No	+++	++	–	30%	IIIC	2	2	TEC	6	0.023	0.001	0.00008
**P21**	50	No	No	PR	No	+++	++	+	40%	IIIC	2	2	TEC	3	0.008	0.001	0.000
**P22**	50	No	No	SD	Yes	+++	+	–	25%	IIIA	2	3	TEC	6	0.020	0.008	0.0002

pCR, pathological complete remission; MPR, major pathological response; ORR, objective response rate; ER, estrogen receptor; PR, progesterone receptor; NAC, neoadjuvant chemotherapy.

Formalin-fixed and paraffin-embedded (FFPE) tissue samples were collected at the time points of diagnosis and surgery. Pre-neoadjuvant chemotherapy (pre-NAC) samples were obtained by core needle biopsy of the breast cancer tissue. Surgically resected specimens were used as paired post-NAC samples.

The study was approved by the ethics review committee of our institution. Written informed consent was obtained from all patients that underwent clinical treatment and biomarker testing. The median follow-up time for clinical outcome was 2.9 years. Clinicopathologic parameters including age, menopausal status, histologic grade, recurrence, follow-up status, and follow-up period were obtained by a thorough review of clinical records.

### Clinical Molecular Typing and Pathological Response Evaluation

To evaluate the molecular subtype classification, the clinical results of immunohistochemistry (IHC) for estrogen receptor (ER), progesterone receptor (PR), and Ki-67 were reviewed. HER2 expression was assessed by IHC and scoring was determined according to the criteria of American Society of Clinical Oncology (ASCO)/College of American Pathologist (CAP) guidelines. Tumors with scores 2+ were further tested by fluorescence *in situ* hybridization (FISH). The level of Ki-67 expression was classified as high versus low with a cutoff point of 20%. ypTN stage was defined according to the guideline by American Joint Committee on Cancer. For this study, pCR was defined as the absence of residual invasive cancer in the breast and axillary nodes with the presence or absence of *in situ* cancer (ypT0/isypN0 or ypT0ypN0), as previously described. Major pathological response (MPR) was defined as ≤10% residual tumor tissue in resected breast and lymph node tissue (15). Objective response rate (ORR) includes Complete Response (CR) and Partial Response (PR) cases, which refers to the proportion of patients whose tumor shrinks to a certain amount and remains for a certain period of time. The effect of NAC was evaluated according to RECIST 1.1.

### Histopathologic Evaluation of Tumor Sections by Light Microscopy

Post-NAC surgical specimens, as well as pre-NAC biopsy samples, were used for FFPE block preparation. Tumor sections sliced from FFPE blocks were subjected to hematoxylin and eosin (H&E) staining. Tumor cells and infiltrating lymphocytes were routinely reviewed by two pathologists. Two to three sections of each tumor specimen were assessed, and the section with highest cancer cell content were subjected to CD3 staining and multiplex IHC.

The percentage of viable tumor cells (averaged across all sections) was reported for each patient, as reported previously (15, 16). Herein, intratumoral TILs or T cells are defined as lymphocytes or T cells in tumor cell aggregates (tumor core) having cell-to-cell contact with no intervening stroma and directly interacting with carcinoma cells, while stromal TILs are located dispersed in the stroma between the carcinoma cells and do not directly contact carcinoma cells (12).

### Multiplex Immunofluorescence Staining for CD4, CD8, PD1, TIM3, and Cytokeratins and Immune Cell Infiltration Analysis

Matched pre-NAC and post-NAC tumor samples were subjected to fluorescent multiplex IHC. Tissue sections 4 μm thick were stained using the PANO 7-plex IHC kit (Cat. #0004100100, Panovue, Beijing, China), which enables the simultaneous visualization of six markers in the same section. Briefly, antigen retrieval was performed with boiling in antigen retrieval solution AR9 (pH 9). Blocking was performed using the Antibody Blocking Solution (Panovue, Cat. #0018001120) for 15 min, followed by incubation with the primary antibodies: TIM3 (CST45208, Cell Signaling Technology, Inc., MA, USA; diluted at 200×), CD8A (CST70306, CST; diluted at 200×), PD1 (CST43248, CST; diluted at 100×), PanCK (CST4545, CST; diluted at 400×), and CD4 (BX22300130; diluted at 2000×). The sections were incubated with the primary antibodies for 30 min at room temperature. Subsequently, the sections were incubated with anti-mouse or anti-rabbit HRP-conjugated Polymer (Panovue, Cat. #0013001010) at room temperature for 15 min, followed by incubation with TSA Opal fluorophores (PPD 520, PPD 540, PPD 570, PPD 620, PPD 650, and PPD 690) for 10 min. After each cycle of staining, the antibody–TSA complex was removed using AR solution (pH 9) and boiling. After staining, all slides were counterstained with DAPI for 5 min and mounted in ProLong Diamond Antifade Mountant (Thermo Fisher).

To obtain multispectral images, the stained slides were scanned using the PerkinElmer Mantra System (or Polaris System, Waltham, Massachusetts, USA). A spectral library required for multispectral unmixing was established using the inForm image software (inForm 2.4.0 PerkinElmer, Waltham, Massachusetts, USA); reconstructed images of each section were obtained using the spectral library. Image analyses using inForm software were performed to determine the recognition and levels of CD4^+^, CD8^+^, PD1^+^, TIM3^+^, PD1^+^CD8^+^, TIM3^+^CD8^+^, PD1^+^CD4^+^, and TIM3^+^CD4^+^ T cells. The percentages of CD4^+^, CD8^+^, and PD1^+^CD8^+^ cells were calculated as the ratio of the number of CD4^+^ or CD8^+^ or PD1^+^CD8^+^ cells over all nucleated cells summed from at least eight selected view fields on each tumor section slide. The denominator is the number of all nucleated cells in all view fields. Clinical endpoints were blind to data collector and statistician before statistical analysis.

### Assessment of CD3^+^ T-Cell Infiltration by IHC and Automatic Scoring

IHC staining for CD3 was conducted using the Dako Omnis autostainer. Briefly, tissue sections 4 µm thick were boiled and then subjected to dewaxing, rehydrating, and antigen retrieval, followed by incubation with an anti-CD3 primary antibody (Dako Omnis, polyclonal rabbit anti-human, Santa Clara, CA, USA). For signal visualization, the EnVision FLEX^+^ High pH (Link) system was used, following the manufacturer’s instructions. The sections were manually mounted in neutral resin for observation under a light microscope. Whole-slide images were acquired using the light microscope on the Mantra System (PerkinElmer, Waltham, Massachusetts, USA). Images were used to quantify the CD3 signal, and the T-cell levels were calculated using the inForm automated image analyses software (PerkinElmer, Waltham, Massachusetts, USA). Briefly, CD3^+^ cells were recognized by machine-learning-based classification according to CD3 staining signal and the percentage was calculated as the number of CD3^+^ cells divided by the total number of nucleated cells in all view fields.

### Testing of Tumor Mutational Burden

Fresh frozen cancer tissues were sliced for pathological assessment. Samples with tumor content more than 60% were subject to further DNA extraction using AllPrep DNA/RNA Mini Kit (Qiagen). Four hundred nanograms of genomic DNA was broken down into 100- to 500-bp fragments measured by Agilent 2100 Bioanalyzer and used for sequencing library construction. Target sequences of 1021 genes at length of 1.07 Mb were then captured using customized reagents and amplified by PCR. After purification and quantification, Target DNA library was sequenced on Gene^+^Seq 2000 platform (Illumina, Inc., USA). Double end sequencing was conducted. Read length was set at 150 bp and depth was 150X. Sequenced results were aligned and mapped to reference genome sequence of GRCh38. TMB calculation is consistent with the traditional method. During the technical validation process, this panel based TMB estimates had good correlation with whole exome sequencing (WES) method. TMB (tumor mutation burden) was defined as the number of non-synonymous mutations (VAF ≥ 0.05) plus common driver gene mutations per megabase of DNA.

### TCR Library Construction and Sequencing

Samples with tumor content of more than 60% were subject to further RNA extraction using AllPrep DNA/RNA Mini Kit (Qiagen). TCR sequence library was constructed using iRepertoire on Cassette Kit iR-TCR Reagent Systems 2.0 (iRepertoire, Inc. 601 Genome Way, Suite 3005, Huntsville, AL 35806). Two steps of PCR procedures were performed. Amplification primers for first run PCR were targeting specific regions of TCR-V and TCR-C. Primers for 2nd PCR were universal containing sequencing primers as follows:

Universal primer A: 5’AATGATACGGCGACCACCGAGATCTACACTCTTTCCCTACACGACGCTCTTCCGATCT-3’;

Universal primer B: 5’CAAGCAGAAGACGGCATACGAGATCGGTCTCGGCATTCCTGCTGAACCGCTCTTCCGATCT-3’.

RNA library was sequenced on Illumina MiSeq platform using Illumina MiSeq Reagent v2500 (MS-102-2003). Results of sequences of regions of V, D, J, and C of TCR were aligned and mapped. CDR3 sequence was generated and metrics of TCR D50, TCR diversity index, and TCR entropy were calculated. D50 is a quantitative measure of the degree of diversity of T cells within a sample. Calculation of D50 is described as in the kit instruction. The D50 is the percent of dominant and unique T-cell clones that account for the cumulative 50% of the total CDR3s counted in the sample. The more diverse a library, the closer the value will be to 50. The equation of D50 is as follows:

D50 = (No. of uCDR3s that make up 50% of the total reads * 100)/No. of uCDR3s (or 10,000 if unique CDR3s are above 10,000).

### Statistical Analyses

All statistical analyses were performed using the SPSS 22.0 software. Continuous variables were described as the mean value ± standard error. TMB values, TCR metrics, and T-cell percentages in pre-NAC and post-NAC samples were compared using the Wilcoxon rank test, while the Mann–Whitney test and Kruskal–Wallis test was used for independent samples. Kaplan–Meier survival curve was plotted for disease-free survival (DFS) and comparison between groups was by log rank test. *p* < 0.05 was considered statistically significant.

## Results

### TMB Is Higher in Non-pCR Versus pCR, Non-MPR Versus MPR, and Non-ORR Versus ORR Patients

**Figure 1:**
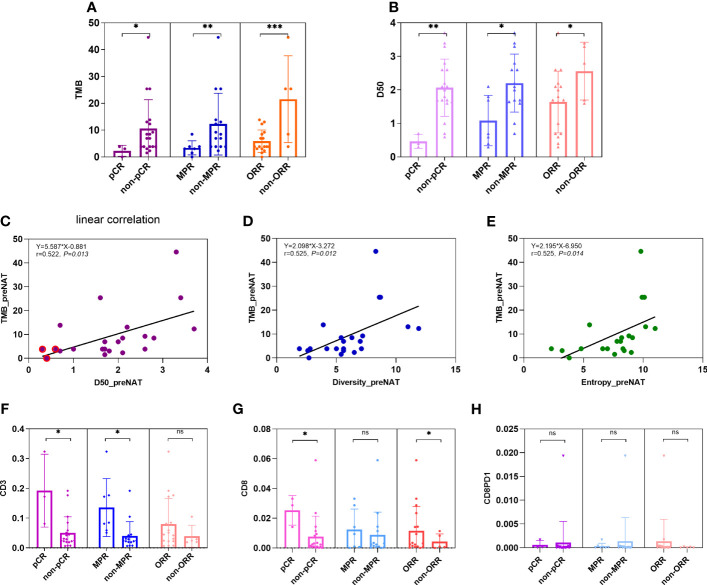
Comparison of TMB, TCR D50, and percentages of CD3^+^, CD8^+^, and PD1^+^CD8^+^ T cells in pre-NAC tumors between groups of pCR versus non-pCR. **(A)** Average TMB is higher in tumors of non-pCR patients than in pCR patients (10.6 vs. 2.3; *p* = 0.043), higher in tumors of non-MPR than MPR patients (12.3 vs. 3.4, *p* = 0.007), and higher in tumors of non-ORR than ORR patients (21.5 vs. 5.7, *p* = 0.001). **(B)** Average TCR D50 is higher in tumors of non-pCR patients than in pCR patients (2.07 vs. 0.47; *p* = 0.010), higher in tumors of non-MPR than MPR patients (2.20 vs. 1.08, *p* = 0.018), and higher in tumors of non-ORR than ORR patients (2.56 vs. 1.64, *p* = 0.034). **(C–E)** Linear correlation was observed between TMB and metrics of TCR like D50 (*y* = 5.587*x* − 0.881; *r* = 0.522, *p* = 0.013), diversity index (*y* = 2.098*x* − 3.272; *r* = 0.525, *p* = 0.012), and entropy (*y* = 2.195*x* − 6.950; *r* = 0.525, *p* = 0.014). **(F)** The percentages of CD3^+^ cells were all higher in pCR versus non-pCR patients (14.35% vs. 5.87%, *p* = 0.050), in MPR vs. non-MPR patients (9.27% vs. 5.98%, *p* = 0.041), but not significantly different between ORR versus non-ORR patients (7.94% vs. 3.94%, 0.347). **(G)** The percentages of CD8^+^ cells were higher in pCR versus non-pCR patients (2.53% vs. 0.75%, *p* = 0.025), numerically but not statistically higher in MPR versus non-MPR patients (1.21% vs. 0.14%, *p* = 0.573), and in ORR versus non-ORR patients (1.16% vs. 0.41%, *p* = 0.034). **(H)** The percentages of CD8^+^PD1^+^ cells were numerically but not statistically lower in pCR versus non-pCR patients (0.07% vs. 0.11%, *p* = 0.072), MPR versus non-MPR patients (0.03% vs. 0.21%, *p* = 0.858), and ORR versus non-ORR patients (0.13% vs. 0.007%, *p* = 0.229). ns, not significant; *p < 0.05; **p < 0.01; ***p < 0.001.

In our study, TMB was defined as the number of non-synonymous mutations plus common driver gene mutations per megabase length of DNA. Tumor mutation burden (TMB) can be detected by next-generation sequencing of a panel of genes with targeted DNA sequence length of more than one megabase. Here we used the panel-based NGS of 1,021 genes with a target length of ~2.1 megabase. We compare TMB values of tumors in patients of pCR versus non-pCR, MPR versus non-MPR, and ORR versus non-ORR in neoadjuvant setting. Average TMB is higher in tumors of non-pCR patients than in pCR patients (10.6 vs. 2.3; *p* = 0.043), higher in tumors of non-MPR than MPR patients (12.3 vs. 3.4, *p* = 0.007), and higher in tumors of non-ORR than ORR patients (21.5 vs. 6.0, *p* = 0.001) (**Figure 1A**).

### TCR Clonality Is Higher in pCR Patients Than in Non-pCR Patients, Correlating With TMB

D50 is a quantitative measure of the degree of diversity of T cells within a sample. The D50 is the percent of dominant and unique T-cell clones that account for the cumulative 50% of the total CDR3s count in the sample. The more diverse a library, the closer the value will be to 50. In our study, the average D50 is higher in tumors of non-pCR patients than in pCR patients (2.07 vs. 0.47; *p* = 0.010), higher in tumors of non-MPR than MPR patients (2.20 vs. 1.08, *p* = 0.018), and higher in tumors of non-ORR than ORR patients (2.56 vs. 1.64, *p* = 0.034) (**Figure 1B**).

Linear correlation was observed between TMB and metrics of TCR like D50 (*y* = 5.587*x* − 0.881; *r* = 0.522, *p* = 0.013), diversity index (*y* = 2.098*x* − 3.272; *r* = 0.525, *p* = 0.012), and entropy (*y* = 2.195*x* − 6.950; *r* = 0.525, *p* = 0.014) (**Figures 1C–E**).

### CD3^+^ and CD8^+^ T Cells Are More Present in pCR Patients Than in Non-pCR Patients

Effector immune cells, especially CD3^+^ or CD8^+^ T cells, have been reported as predictive or prognostic biomarkers in cancers. In our study, we reviewed HE slides for single-plex CD3^+^ cells, and multi-plex testing of markers CD4, CD8, PD1, and TIM3. Here, we showed the percentages of CD3^+^ cells and CD8^+^ cells were all higher in pCR versus non-pCR patients (14.35% vs. 5.87%, *p* = 0.050; 2.53% vs. 0.75%, *p* = 0.025) and in MPR vs. non-MPR patients (9.40% vs. 5.98%, *p* = 0.041; 1.21% vs. 0.14%, *p* = 0.573) (**Figures 1F, G**).

CD8^+^ T-cell percentages were higher in ORR versus non-ORR patients (1.16% vs. 0.42%, *p* = 0.034). PD1^+^CD8^+^ cells were numerically lower in pCR versus non-pCR patients (0.07% vs. 0.11%, *p* = 0.072) and MPR versus non-MPR patients (0.03% vs. 0.21%, *p* = 0.858) (**Figure 1H**).

**Figure 2:**
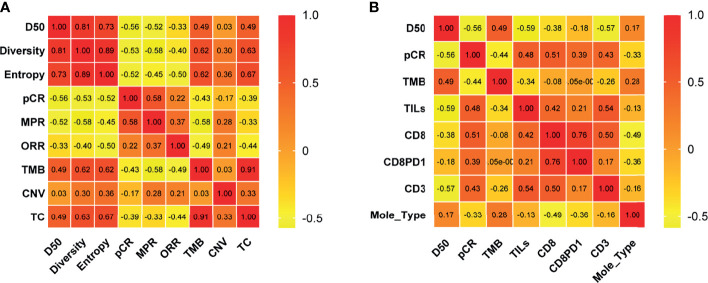
Correlation of molecular and clinical response variables. Correlation coefficients between two variables among TCR D50, Diversity index, Entropy, pCR, MPR, ORR, TMB, CNV, and T cell percentages in tissues of pre-NAC samples **(A)** and among D50, pCR, TMB, TILs, CD8, CD8PD1, CD3 and molecular types **(B)** were listed. The number in the color chart is the correlation coefficient. pCR, pathologic complete response; MPR, major pathological response; ORR, Objective Response Rate; TMB, tumor mutational burden; CNV, Copy number variations; TC, TMB and CNV; Mole_Type, Molecular typing.

Correlation coefficients of all factors were calculated. We found that TCR metrics and TMB and clinical efficacy factors had significant correlation (**Figure 2A**), and percentages of CD3^+^ T cells had significant association with TCR D50 and pCR (**Figure 2B**).

### Dynamic Changes of TMB and TCR Index Before and After Neo-Adjuvant Therapies

**Figure 3:**
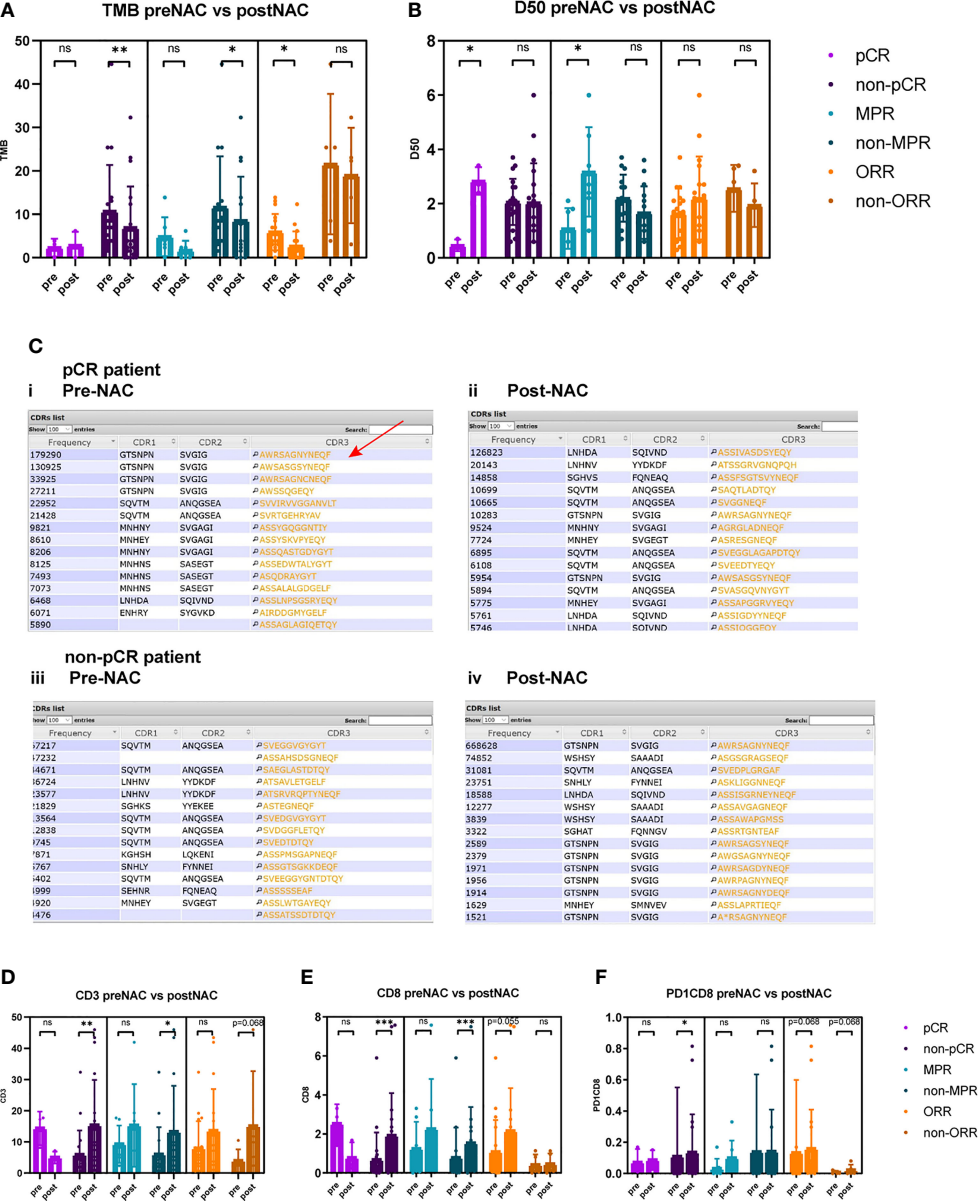
Comparison of TMB, TCR D50, CD3^+^, CD8^+^, and PD1^+^CD8^+^ cells between pre-NAC and post-NAC tumor tissues. **(A)** TMB reduced significantly after neoadjuvant therapy in non-pCR patients (10.6 vs. 6.8, *p* = 0.010), in non-MPR patients (12.3 vs. 8.5, *p* = 0.041), and in ORR patients (6.0 vs. 2.6, *p* = 0.017). TMB was not significantly different between pre-NAC and post-NAC tumors or non-cancerous residual tissues in pCR patients (2.3 vs. 2.8, *p* = 0.879), in MPR patients (3.4 vs. 1.6, *p* = 0.176), and in non-ORR patients (21.5 vs. 18.9, *p* = 0.500). **(B)** TCR D50 index significantly increased post-NAC versus pre-NAC in pCR patients (0.467 vs. 2.883, *p* = 0.028) and in MPR patients (1.086 vs. 3.171, *p* = 0.028), but not significantly change after NAC in non-pCR patients (D50 2.066 vs. 2.037, *p* = 0.658), and in non-MPR patients (2.203 vs. 1.667, *p* = 0.125), and in both ORR (1.638 vs. 2.206, *p* = 0.332) and non-ORR patients (2.560 vs. 1.940, *p* = 0.343). **(C)** Top 15 CDR3 motifs in pre-NAC tumors (i, iii) and post-NAC residual non-cancerous or cancerous tissues (ii, iv) of pCR and non-pCR patients. CDR3 motif is the major element recognizing the tumor specific mutated antigens. The CDR3 peptide AWRSAGNYNEQF is the highly expressed TCR in pre-NAC samples of pCR patients (Red arrow) and in post-NAC samples of non-pCR patients. **(D)** In non-pCR patients and non-MPR patients, the percentages of CD3^+^ T cells significantly increased after NAC (5.9% vs. 15.3%, *p* = 0.003; 5.9% vs. 13.2%, *p* = 0.019). **(E)** In non-pCR patients and non-MPR patients, the percentages of CD8^+^ T cells significantly increased after NAC (0.8% vs. 1.9%, *p* = 0.001; 0.1% vs. 1.5%, *p* = 0.001). **(F)** In non-pCR patients, the percentages of PD1^+^CD8^+^ T cells significantly increased after NAC (0.11% vs. 0.13%, *p* = 0.025). ns, not significant; *p < 0.05; **p < 0.01; ***p < 0.001.

Dynamically, the TMB reduced significantly after neoadjuvant therapy in non-pCR patients (10.6 vs. 6.9 *p* = 0.010) but not in pCR patients (**Figure 3A**). TCR diversity index increased in pCR patients (D50 0.467 vs. 2.883, *p* = 0.028) (**Figure 3B** and **Supplementary Figures 1A**, **2B**). TCR diversity index did not change significantly in pre-NAC versus post-NAC tissues of non-pCR patients (D50 2.066 vs. 2.037, *p* = 0.658) (**Figure 3B**), but in some particular cases, TCR clonality transformed to a high level (**Supplementary Figures 1A**, **2B**). CDR3 motif is the major element recognizing the tumor-specific mutated antigens. The CDR3 peptide AWRSAGNYNEQF is the highly expressed TCR in pre-NAC samples of pCR patients and in post-NAC samples of non-pCR patients, suggesting that there is a special CDR3 responsive to neoadjuvant therapies (**Figure 3C**).

Notably, in non-pCR tumors, CD3^+^, CD8^+^, and PD1^+^CD8^+^ T-cell percentages significantly increased after neoadjuvant therapy (5.87% vs. 15.31%, *p* = 0.033; 0.75% vs. 1.89%, *p* < 0.001; 0.11% vs. 0.13%, *p* = 0.025) (**Figures 3D–F**).

The comparisons of TMB, D50, CD3^+^, CD8^+^, CD4^+^, PD1^+^, PD1^+^CD8^+^, and other T-cell immune factors between pre-NAC and post-NAC tissues in the general population or patients with different clinical endpoints are shown in **Table 2**.

**Table 2:** Comparison of TMB, D50, and immune factors pre-NAC and post-NAC in general population or patients with different curative effects.

	Non-pCR	pCR	*p*	Non-MPR	MPR	*p*	Non-ORR	ORR	*p*	ALL
**TMB Pre-NAC**	10.639	2.310	**0.043**	12.347	3.409	**0.007**	21.538	5.964	**0.001**	9.503
**TMB Post-NAC**	6.882	2.821	0.699	8.513	1.65	**0.023**	18.923	2.624	**0.000**	6.329
***p***	**0.010**	0.879		**0.041**	0.176		0.500	**0.017**		**0.014**
**D50 Pre-NAC**	2.066	0.467	**0.010**	2.203	1.086	**0.018**	2.560	1.638	**0.034**	1.848
**D50 Post-NAC**	2.037	2.833	0.125	1.667	3.171	**0.031**	1.940	2.206	0.875	2.146
***p***	0.658	**0.028**		0.125	**0.028**		0.343	0.332		0.638
**CD3 Pre-NAC**	0.059	0.143	**0.050**	0.059	0.094	**0.041**	0.039	0.079	0.077	0.070
**CD3 Post-NAC**	0.153	0.050	0.191	0.132	0.153	0.490	0.151	0.135	0.906	0.139
***p***	**0.003**	0.109		**0.019**	0.499		**0.068**	0.177		**0.050**
**CD8 Pre-NAC**	0.008	0.025	**0.025**	0.009	0.012	0.573	0.004	0.012	**0.034**	0.011
**CD8 Post-NAC**	0.019	0.008	0.356	0.0154	0.023	0.535	0.005	0.022	**0.021**	0.017
***p***	**0.000**	0.109		**0.001**	0.735		0.144	**0.055**		**0.021**
**CD4 Pre-NAC**	0.040	0.059	0.166	0.055	0.024	0.410	0.030	0.046	0.693	0.021
**CD4 Post-NAC**	0.039	0.008	0.343	0.031	0.045	0.462	0.038	0.036	0.346	0.036
***p***	0.959	0.028		0.128	0.465		0.655	0.515		0.657
**PD1 Pre-NAC**	0.024	0.004	0.693	0.030	0.005	0.143	0.004	0.024	0.693	0.021
**PD1 Post-NAC**	0.012	0.008	0.752	0.013	0.009	0.705	0.021	0.009	0.480	0.011
***p***	0.575	**0.039**		0.499	0.715		0.180	0.314		0.722
**TIM3 Pre-NAC**	0.008	0.001	0.324	0.010	0.002	0.107	0.001	0.008	0.324	0.007
**TIM3 Post-NAC**	0.003	0.0004	0.343	0.002	0.006	0.186	0.002	0.003	0.814	0.003
***p***	0.241	0.635		**0.043**	0.465		0.655	0.214		0.213
**PD1CD8 Pre-NAC**	0.001	0.0007	**0.072**	0.001	0.0003	0.858	0.00007	0.001	0.229	0.001
**PD1CD8 Post-NAC**	0.001	0.0009	1.000	0.0014	0.0010	0.237	0.00023	0.002	0.108	0.002
***p***	**0.025**	0.593		0.140	0.128		0.068	0.068		**0.027**
**PD1CD4 Pre-NAC**	0.004	0.001	0.423	0.006	0.000	0.158	0.001	0.004	1.000	0.004
**PD1CD4 Post-NAC**	0.003	0.000	0.114	0.003	0.001	0.705	0.008	0.001	0.099	0.002
***p***	0.799	**0.036**		0.398	0.465		0.180	0.173		0.657
**TIM3CD8 Pre-NAC**	0.0006	0.000	0.335	0.001	0.000	0.074	0.000	0.001	0.335	0.0005
**TIM3CD8 Post-NAC**	0.0002	0.000	0.202	0.000	0.000	0.849	0.000	0.000	0.634	0.0002
***p***	0.953	0.108		0.345	0.109		0.180	0.735		0.477
**TIM3 CD4 Pre-NAC**	0.004	0.0003	1.000	0.005	0.000	**0.045**	0.000	0.004	0.689	0.003
**TIM3 CD4 Post-NAC**	0.001	0.000	0.114	0.001	0.002	0.571	0.001	0.001	0.480	0.001
***p***	0.575	0.083		0.091	0.273		0.180	0.314		0.953
**CD4/CD8 Pre-NAC**	5.256	5.145	0.197	6.392	2.929	0.734	3.025	5.680	0.519	5.237
**CD4/CD8 Post-NAC**	1.682	1.806	0.343	1.778	1.545	0.571	3.744	1.238	**0.034**	1.693
***p***	0.086	0.092		**0.063**	0.593		0.180	**0.025**		**0.047**

NAC, neo-adjuvant chemotherapy; Clinical response was evaluated according to RECIST (the Response Evaluation Criteria in Solid Tumors) 1.1; CR, complete response; PR, partial response; SD, stable disease; PD, progressive disease; MPR, 10% or less percentage of residual viable tumor cells after neoadjuvant therapy; ER, estrogen receptor; PR, progesterone receptor; HER2, human epidermal growth factor receptor 2; TNBC, triple-negative breast cancer. The bold values were those p values less than 0.05.

### Top Five Mutated Genes in Pre-NAC and Post-NAC Samples

**Figure 4:**
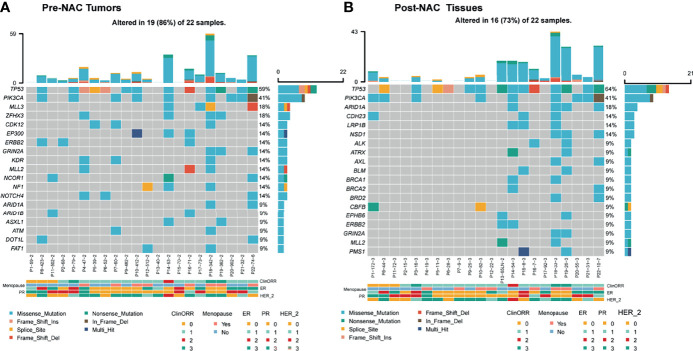
Landscape of genomic alterations in pre-NAC tumors and post-NAC residual non-cancerous tissues (pCR) or cancerous tissues (non-pCR). **(A)** In pre-NAC tumors, the top 15 mutated genes were *TP53, PIK3CA, MLL3, ZFHX3, CDK12, EP300, ERBB2, GRIN2A, KDR, MLL2, NCOR1, NF1, NOTCH4, ARID1A*, and *ARID1B*. **(B)** In post-NAC residual non-cancerous tissues (pCR) or cancerous tissues (non-pCR), the top 15 mutated genes were *TP53, PIK3CA, ARID1A, CDH23, LRP1B, NSD1, ALK, ATRX, AXL, BLM, BRCA1, BRCA2, BRD2, CBFB*, and *EPHB6*. cliniORR, clinical objective response rate; menopause, menopause; ER, estrogen receptor; PR, progesterone receptor; HER-2_, human epidermal growth factor receptor 2. Missense_mutation, nonsense mutation; frame_shift_ins, frame shift insertion mutation; spice_site, splicing mutation; frame_shift del, Frameshift deletion mutation, nonsense mutation, nonsense mutation, in frame del, frameshift deletion mutation, multi hit, multiple site mutation of a same gene; ClinORR 0: Complete Response (CR), 1: Partial Response (PR), 2: Stable Disease (SD), 3: Progressive Disease (PD); ER, PR, HER_2, 0: Negative, 1: +, 2: ++, 3: +++.

Landscape of genomic alterations of breast cancers has been reported previously. Here in this study, the top five mutated genes are *TP53, PIK3CA, MLL3, ZFHX3*, and *CDK12* in pre-NAC samples and *TP53, PIK3CA, ARID1A, CDH23*, and *LRP1B* in post-NAC samples (**Figure 4**), which were similar to other studies (17). Genes with significant loss of genomic alterations after neoadjuvant treatment include *ZFHX3*, *CDK12*, and *EP300*.

### High TCR Clonality and Percentage of CD8^+^ and PD1^+^CD8^+^ T Cells Are Associated With Disease-Free Survival

**Figure 5:**
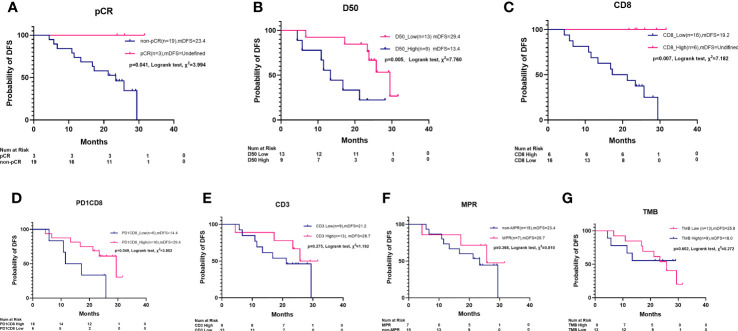
Kaplan–Meier survival plot of disease-free survival by pCR, MPR, TCR D50, PD1^+^CD8^+^ cells, CD3^+^ cells, CD8^+^ cells, and TMB. **(A)** pCR patients had a longer median disease-free survival (mDFS) than non-pCR patients (NR vs. 23.4 m, Log rank test, *χ* ^2^ = 3.994, *p* = 0.041). **(B)** A longer mDFS was observed in patients with high TCR clonality than those with low TCR clonality (29.4 m vs. 13.4 m, Log rank test, *χ* ^2^ = 6.660, *p* = 0.009). **(C)** A longer mDFS was observed in patients with high-level CD8^+^ cells than those with low-level CD8^+^ cell (NR vs. 19.2 m, Log rank test, *χ* ^2^ = 7.182, *p* = 0.007). **(D)** A longer mDFS was observed in patients with high-level PD1^+^CD8^+^ cells than those with low level PD1^+^CD8^+^ cells (29.4 m vs. 14.4 m, Log rank test, *χ* ^2^ = 3.852, *p* = 0.049). **(E)** mDFS was numerically but not statistically longer in patients with high-level CD3^+^ cells than those with low-level CD3^+^ cells (28.7 m vs. 21.3 m, *χ* ^2^ = 1.192, *p* = 0.275). **(F)** Not statistically different mDFS was found between MPR patients and non-MPR patients (25.7 m vs. 23.4 m, Log rank test, *χ* ^2^ = 0.810, *p* = 0.368). **(G)** Not statistically different mDFS was found between high TMB and low TMB patients (25.8 m vs. 18.0 m, Log rank test, *χ* ^2^ = 0.272, *p* = 0.602).

In addition to pCR (**Figure 5A**), high clonality of TCR (D50) and the percentage of infiltrating CD8^+^ and PD1^+^CD8^+^ T cells are associated with DFS by KM survival analysis (**Figures 5B–D**). A longer DFS was observed in patients with lower D50 versus higher D50 (29.4 vs. 13.4 months, *p* = 0.005), in patients with more CD8^+^ T cells than those with low CD8^+^ cells (NR vs. 19.2 months, *p* = 0.007), and in patients with more PD1^+^CD8^+^ T cells than in those with low PD1^+^CD8^+^ cells (29.4 vs. 14.4 months, *p* = 0.049). Difference of DFS was not observed between groups of high versus low CD3^+^ T cells, MPR versus non-MPR, or high versus low TMB (**Figures 5E–G**).

## Discussion

Breast cancer is the most frequently diagnosed cancer and the leading cause of cancer-related death in women globally (1–3). Neo-adjuvant chemotherapy (NAC) is routine standard of care for women with high-risk, early-stage BC by improving both radical surgery and breast-conserving surgeries and reducing axillary lymph node metastasis (18, 19). However, not all patients unanimously get improved clinical outcome. Thus, one of clinical challenges is how to precisely select the beneficiary patients for NAC.

Several categories of predictive biomarkers in pre-NAC tumors were recently investigated, including clinical genotyping, pCR, immune biomarkers, and so on. In breast tumors it is worth simultaneously studying the relationship between multiple categories of immune biomarkers and clinical outcomes. Sophisticated technologies like multi-plex immunofluorescent cytochemistry, next-generation sequencing-based TMB, TCR repertoire analysis, single-cell RNA sequencing, and spatial transcriptome sequencing have been developed recently. These technologies make it possible to comprehensively study categories of biomarkers in one tumor. Herein, we reported comprehensive biomarkers of TCR, TMB, and T cells in the same specimens of breast cancer tissues before and after NAC.

Our study showed that TMB is consistently higher in non-pCR versus pCR, non-MPR versus MPR, and non-ORR versus ORR patients, suggesting that TMB could predict the efficacy in neoadjuvant chemotherapy setting of breast cancer. In pCR patients, the lower TMB may suggest only a few somatic mutations account for the immunogenicity of these tumors, which are also responsive to NAC.

Correspondingly, in this cohort, TCR clonality is higher in pCR patients than in non-pCR patients. The metric of TCR D50 is a quantitative measure of the degree of diversity of T cells within a sample. The TCR D50 value is the percentage of dominant and unique T-cell clones that account for the cumulative 50% of the total CDR3s count in the sample. The more diverse a library, the closer the value will be to 50. In our study, average D50 is higher in tumors of non-pCR patients than in pCR patients, and higher in tumors of non-MPR than MPR patients, and higher in tumors of non-ORR than ORR patients as well. In addition, TCR clonality correlates with TMB significantly. Linear correlation was observed between TMB and metrics of TCR like D50, diversity index, and entropy. Similar data were reported previously in lung cancer, showing that TCR entropy was linearly correlated with TMB (20). This notable correlation indicated the interaction between TCR and TMB in a neoadjuvant setting of breast cancers. In the GeparNuevo study of TNBC, high TMB associated with pCR, while in our study, low TMB was found in the three pCR tumors. This may be due to the heterogenous population, more stage III disease and different TMB testing platform in our study. We think that in NAC-responsive patients, a few mutated antigens may sufficiently elicit immune response, inducing clonal expansion of responsive T cells, which further provides basis for the antitumor activity of NAC through enhancing immune killing.

To confirm if immune reactive cells were recruited into TME, we also detected the presence of immune cells by the single-plex IHC and quantitative multiple immunofluorescent cytochemistry (mIFC) method. We observed that CD3 and CD8 are significantly at higher expression levels in pCR patients than non-pCR patients. Effector immune cells, especially CD3^+^ or CD8^+^ T cells, have been reported as predictive or prognostic biomarkers in cancers. In our study, we reviewed HE slides for single-plex CD3^+^ cells and multi-plex testing of CD4^+^ and CD8^+^ T cells and immune checkpoint markers PD1 and TIM3. Here, we found that CD4^+^ cell percentages were not significantly different in patients of pCR versus non-pCR, MPR versus non-MPR, or ORR versus non-ORR (**Table 2**). However, the percentages of CD3^+^ cells and CD8^+^ cells were all higher in pCR versus non-pCR patients, in MPR versus non-MPR patients. CD8^+^ T cells were higher in ORR versus non-ORR patients. PD1^+^CD8^+^ cells were numerically lower in pCR versus non-pCR patients, and MPR versus non-MPR patients. Collectively, these data suggested that reactive immune cells were also frequently present in NAC-responsive tumors in a neoadjuvant setting.

We further looked at the dynamic changes of TMB and TCR heterogeneity before and after NAC-treated breast cancers. The TMB reduced significantly after neoadjuvant therapy in non-pCR patients but without TCR index change, while TCR diversity index increased in pCR patients. Notably, CDR3 motif is the major element recognizing the tumor-specific mutated antigens. The CDR3 peptide AWRSAGNYNEQF is the highly expressed TCR in pre-NAC samples of pCR patients and in post-NAC samples of non-pCR patients, suggesting that there is a special CDR3 motif responsive to neoadjuvant therapies. The sequence of these top CDR3 motifs and their functional and predictive roles in BC need to be explored in a future study.

In addition, in non-pCR tumors, CD3^+^ and CD8^+^ cells significantly increased after neoadjuvant therapy, suggesting that even without significant clinical benefit, the immune microenvironment can be improved through NAC by recruiting more effective immune cells. This may be due to the fact that chemotherapy could induce the death and antigen release of cancer cells and further increase the immune cells’ infiltration by chemotaxis of upregulated expression of intratumoral chemokines (21, 22). Notably, in our study, PD1^+^CD4^+^ cells were found reduced in post-NAC in comparison with pre-NAC tumors. TIM3^+^CD4^+^ cell percentages were lower in MPR versus non-MPR tumors, but were not significantly different between pCR and non-pCR pre-NAC tumors. Furthermore, studies also showed that certain lymphocyte populations may have specific roles in different molecular subtypes of breast cancer during the response to neoadjuvant treatments. Thus, detailed analysis of lymphocyte subpopulations can provide critical information on the formation and dynamic changes of immune status in tumors (23). Breast cancers have already been routinely treated based on the molecular genotyping of hormone receptors and HER2. Landscape of genomic alterations of breast cancers has been reported previously (24, 25). Genomic alterations of other driver genes in breast cancers were also reported in association with prognosis. Here, we tested the mutations by NGS of 1,021 genes; in this study, the top five mutated genes are *TP53, PIK3CA, MLL3, ZFHX3*, and *CDK12* in pre-NAC samples and *TP53, PIK3CA, ARID1A, CDH23*, and *LRP1B* in post-NAC samples, which were similar to other studies. Genes with significant loss of genomic alterations after neoadjuvant treatment include *ZFHX3*, *CDK12*, and *EP300*, suggesting that, in addition to pro-proliferation (*PIK3CA*), genomic instability (*TP53*), cell cycle (*CDK12*), chromatin remodeling, and epigenetic modification (*ARID1A*, *EP300*) are also prevalent in breast cancer. Among the recognized mutated genes, *TP53* and *PIK3CA* mutations seemed to be early events and clonal present in both pre-NAC and post-NAC lesions. Clones harboring other mutated genes like *CDK12*, *NOTCH4*, and *KDR* might be more sensitive to NAC treatment, which were not detected in post-NAC residual tumors. The persistent mutations of PIK3CA and other genes can be exploited to develop combinatorial targeted therapy with chemotherapy or immunotherapy, as previous data showing PIK3CA inhibition plus hormone receptor inhibition had activity in some patients (26).

Recently, ongoing trials showed that promising PD1 inhibitor in combination chemotherapy greatly increases the pCR rate in neoadjuvant setting of breast cancers (27). Tumor mutational burden and immune infiltration had also been shown as independent predictors of response to neoadjuvant immune checkpoint inhibition in early TNBC in the GeparNuevo trial (28). In the study, the used immune gene expression signature included a list of genes including CXCL9, CCL5, CD8A, CD80, CXCL13, IDO1, PDCD1, CD274, CTLA4, and FOXP3. The predictive value of TMB was found significant both for immune checkpoint inhibition with chemotherapy and for chemotherapy alone. It was also reported that high expression of immune-related gene signatures, including cytotoxic molecules, T-cell receptor signaling components, cytokines related to T helper cell type 1 (Th1), and B-cell markers was associated with a pCR in TNBC (29). Expression of NFKB1, TRAF1, and CXCL13 genes, in particular, was significantly correlated with a longer DFS rate. Patients who failed to achieve a pCR showed increased expression of genes related to neutrophils.

In HER2+ BC, immune molecules were also reported in association with pCR in neoadjuvant setting (30). In the NeoALLTO trial, in addition to high levels of ERBB2/HER2 and low levels of ESR1, high expression of immune and stroma gene signatures were significantly associated with higher and lower pCR rates, respectively, and should be further explored as candidate predictive markers (31). Use of patterns of TRBV genes potentially provide information about the association with response to dual HER2 blockade beyond immune gene signatures (32). High use of TRBV11.3 or TRBV4.3, TRBV6.3, and TRBV7.2 identifies patients who have a better response to dual HER2 targeted therapy.

In our study, we had not treated patients with immune checkpoint inhibitor, so we could not directly infer the efficacy of immune therapy in these patients. However, the dynamic changes of immune cell infiltration and genetic profiles after NAC suggested that the immune therapies could probably benefit these patients, because the increasing infiltration of T cells into tumor core induced by NAC is a positive signal for anticancer activity.

Finally, we observed that, in addition to pCR, high clonality of TCR and CD8 expression are associated with better DFS. A longer DFS was observed in patients with lower D50 than those with higher D50, in patients with higher infiltration of lymphocytes than those with low TILs, or in patients with more CD8^+^ T cells than those with low CD8^+^ cells. We also calculated the overall survival (OS) but found no significant difference between subgroups of pCR versus non-pCR, MPR versus non-MPR, high D50 versus low D50, or high versus low infiltration of immune cells (**Supplementary Figure 3**), which might be due to not long and immature follow-up. These results suggested that NAC-associated immune microenvironment modification could be translated into longer benefit of DFS in a neo-adjuvant setting of chemotherapy.

This study had several limitations. It was a single-center study with a relatively small sample size, which may limit statistical significance. Sample size will be greater in a future study. We did not assess the relationship between immune markers and the final endpoint of OS due to limited follow-up data. Our analyses were restricted to CD4, CD8, PD1, and TIM3; several other markers of critical immune cell subtypes have been established, including FoxP3, CD127, CD68, CD103, and CD19, but these were not examined due to the limited number of markers that can be stained in the same run using mIFC and short of sufficient pre-NAC small biopsy samples. Functional status of infiltrating T cells also needs to be further studied as reported to correlate to the clinical outcome of breast cancers (28, 33). Single-cell RNA sequencing can help to generate an immune map of continuous T-cell activation and differentiation states as reported (34).

## Conclusions

In summary, our results demonstrated that TCR index and TMB have significant interaction and may guide neo-adjuvant treatment in operable breast cancers. Response to NAC in tumors with high TCR clonality may be attributable to high infiltration and expansion of TILs or CD8-positive effector cells. Infiltration of T cells into TME by NAC may predispose tumors to PD1/PDL1 inhibition alone or in combination with NAC. Persistent PIK3CA mutations in pre-NAC and post-NAC tissues may also warrant targeted therapy in combination with chemotherapy or immunotherapy. However, these reflections merit further investigation.

## Data Availability Statement

The original contributions presented in the study are included in the article/**Supplementary Material**. Further inquiries can be directed to the corresponding authors.

## Ethics Statement

The studies involving human participants were reviewed and approved by the Medical Ethics Committee of Affiliated Cancer Hospital & Institute of Guangzhou Medical University. The patients/participants provided their written informed consent to participate in this study. Written informed consent was obtained from the individual(s) for the publication of any potentially identifiable images or data included in this article.

## Author Contributions

Conceptualization: HLL, XZ, and JH. Data curation (e.g., statistical analysis, biostatistics, computational analysis): HLL and XZ. Acquisition of data (acquired and managed patients, provided facilities, etc.): HLL, JQH, HSL, XA, WBG, LJ, JM, CW, WH, WLG, MJ, HX, ZY, and YZ. Pathological evaluation: HLL and XT. Funding acquisition: JQH, XZ, and HLL. Methodology: HLL, XZ, YC, DL, and ZL. Project administration or material support (i.e., reporting or organizing data, and constructing databases): JQH, XZ, HSL, and HLL. Software: HLL. Supervision: JQH and JH. Validation: HLL and XZ. Visualization: HLL. Roles/Writing—original draft and Writing—review and editing: HLL, XZ, and JH. All authors contributed to the article and approved the submitted version.

## Funding

This study was funded by Guangzhou Health S&T Project (No.20191A011097, HLL), Guangzhou S&T Project (No.202102080096, HLL; No.201904010331, JQH), Guangdong Educational Bureau Program (No.2020KTSCX105, JQH), Guangdong Provincial Natural Science Program (No.2019A1515010900, X Zhang), GDPH Dengfeng Program (No. DFJH201903 & KJ012019444 & 8197103306, XZ), Natural Science Foundation of Guangdong Province (2017A030313551, HSL), Natural Science Foundation China (NSFC No.82173202, X Zhang; NSFC No.82072899, HS Li), Guangzhou Municipal Science and Technology Bureau (No.202102010042, HS Li), and Guangzhou High-level Clinical Key Specialty Construction Project and Clinical Key Specialty Project of Guangzhou Medical University (202005).

## Conflict of Interest

Authors LJ and JM were employed by the company Panovue Biological Technology Co. Author CW was employed by the company Geneplus-Shenzhen.

The remaining authors declare that the research was conducted in the absence of any commercial or financial relationships that could be construed as a potential conflict of interest.

## Publisher’s Note

All claims expressed in this article are solely those of the authors and do not necessarily represent those of their affiliated organizations, or those of the publisher, the editors and the reviewers. Any product that may be evaluated in this article, or claim that may be made by its manufacturer, is not guaranteed or endorsed by the publisher.

## Supplementary Material

Supplementary Figure 1 Visualization of TCR clonotypes in tissues of pre-NAC versus post-NAC. Representative visualization plots of TCR clonotypes in tissues of pre-NAC versus post-NAC in a pCR patient **(A)** and non-pCR patient **(B)**. In the tissue microenvironment, the total number of TCR clonotypes increased after NAC in pCR patient, while in non-pCR patient the number decreased suggesting some special clonotypes expanded after NAC in the tumor microenvironment.

Supplementary Figure 2 Calculation of TCR D50 values in samples of pre-NAC versus post-NAC. Representative TCR D50 plots in tissues of pre-NAC versus post-NAC in a pCR patient **(A)** and non-pCR patient **(B)**. In the tissue microenvironment of the pCR patient, the TCR D50 values was 0.7 and 2.4 in pre-NAC and post-NAC tissues respectively. In the tumor microenvironment of the non-pCR patient, the TCR D50 values was 2.2 and 0.4 in pre-NAC and post-NAC tissues respectively.

Supplementary Figure 3 Kaplan Meir survival plot of overall survival by pCR, MPR, TCR D50, PD1^+^CD8^+^ cells, CD3+ cells, CD8^+^ cells, TMB. **(A)**. median overall survival (mOS) was not significantly different between pCR and non-pCR patients (NR vs 33.8m, Log rank test, χ^2^ = 0.345, P=0.557). **(B)**. mOS was not significantly different between low D50 and high D50 patients (NR vs 33.8m, Log rank test, χ^2^ = 0.165, P=0.684). **(C)**. mOS was not significantly in patients with high level versus low level of CD8^+^ cells infiltration (NR vs 33.8m, Log rank test, χ^2^ = 0.409, P=0.523). **(D)**. mOS was not significantly different in patients with high level versus low level of PD1^+^CD8^+^ cells infiltration (33.8m vs NR, Log rank test, χ^2 =^ 0.733, P=0.392). **(E)**. mOS was not significantly different in patients with high level versus low level of CD3^+^ cells infiltration (NR vs 33.8m, Log rank test, χ^2^ = 0.028, P=0.867). **(F)**. mOS was not significantly different between MPR patients and non-MPR patients (NR vs 33.8m, Log rank test, χ^2^ = 1.048, P=0.305). **(G)**. mOS was not significantly different between low TMB and high TMB patients (NR vs 33.8m, Log rank test, χ^2^ = 0.104, P=0.747).

## Abbreviations

pCR, pathological complete response; mIHC, multiplex immunohistochemistry; TILs, tumor-infiltrating lymphocytes; NACs, neoadjuvant chemotherapies; TCRs, T-cell receptors.
