# Supplementary figures and images for: TMB and TCR Are Correlated Indicators Predictive of the Efficacy of Neoadjuvant Chemotherapy in Breast Cancer
Source: Front Oncol. 2021 Dec 7;11:740427. doi: 10.3389/fonc.2021.740427 (PMC8688823; doi:10.3389/fonc.2021.740427)

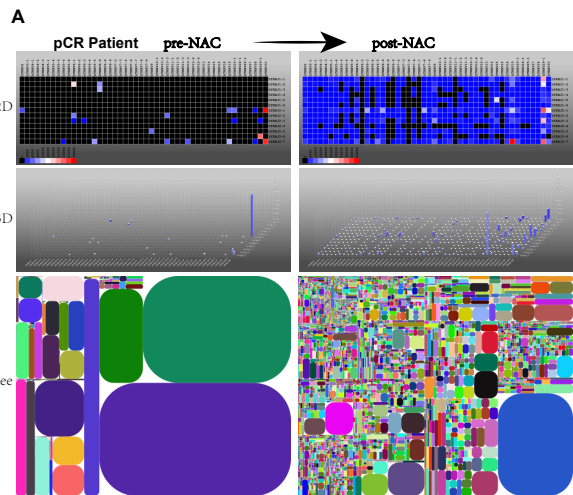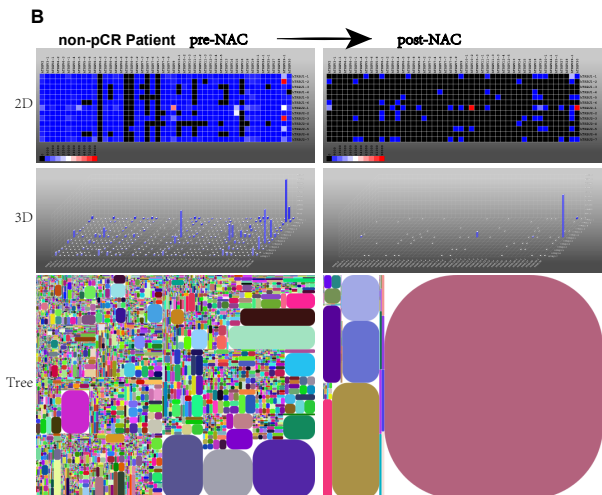

Supplement: Supplementary Figure 1 — Visualization of TCR clonotypes in tissues of pre-NAC versus post-NAC. Representative visualization plots of TCR clonotypes in tissues of pre-NAC versus post-NAC in a pCR patient (A) and non-pCR patient (B). In the tissue microenvironment, the total number of TCR clonotypes increased after NAC in pCR patient, while in non-pCR patient the number decreased suggesting some special clonotypes expanded after NAC in the tumor microenvironment. [file DataSheet_1.pdf]

**A**

pCR patient

pre-NAC

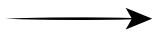

Post-NAC

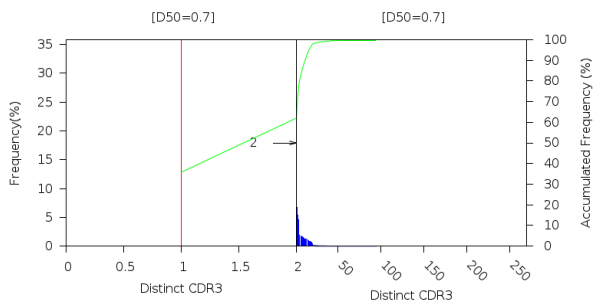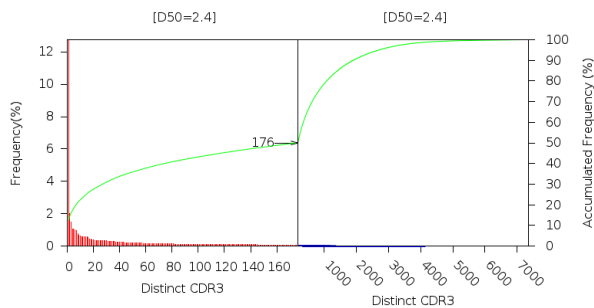

**B**

non-pCR patient

pre-NAC

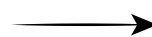

Post-NAC

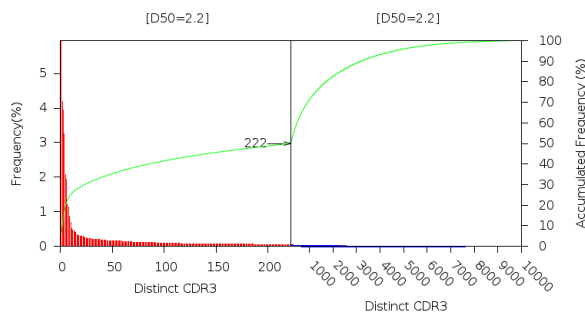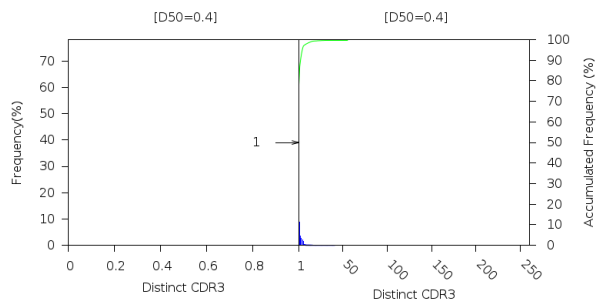

Supplement: Supplementary Figure 2 — Calculation of TCR D50 values in samples of pre-NAC versus post-NAC. Representative TCR D50 plots in tissues of pre-NAC versus post-NAC in a pCR patient (A) and non-pCR patient (B). In the tissue microenvironment of the pCR patient, the TCR D50 values was 0.7 and 2.4 in pre-NAC and post-NAC tissues respectively. In the tumor microenvironment of the non-pCR patient, the TCR D50 values was 2.2 and 0.4 in pre-NAC and post-NAC tissues respectively. [file DataSheet_2.pdf]
