# Supplementary material for: TMB and TCR Are Correlated Indicators Predictive of the Efficacy of Neoadjuvant Chemotherapy in Breast Cancer
Source: Front Oncol. 2021 Dec 7;11:740427. doi: 10.3389/fonc.2021.740427 (PMC8688823; doi:10.3389/fonc.2021.740427)
Supplement: Supplementary Figure 3 — Kaplan Meir survival plot of overall survival by pCR, MPR, TCR D50, PD1+CD8+ cells, CD3+ cells, CD8+ cells, TMB. (A). median overall survival (mOS) was not significantly different between pCR and non-pCR patients (NR vs 33.8m, Log rank test, χ2 = 0.345, P=0.557). (B). mOS was not significantly different between low D50 and high D50 patients (NR vs 33.8m, Log rank test, χ2 = 0.165, P=0.684). (C). mOS was not significantly in patients with high level versus low level of CD8+ cells infiltration (NR vs 33.8m, Log rank test, χ2 = 0.409, P=0.523). (D). mOS was not significantly different in patients with high level versus low level of PD1+CD8+ cells infiltration (33.8m vs NR, Log rank test, χ2 = 0.733, P=0.392). (E). mOS was not significantly different in patients with high level versus low level of CD3+ cells infiltration (NR vs 33.8m, Log rank test, χ2 = 0.028, P=0.867). (F). mOS was not significantly different between MPR patients and non-MPR patients (NR vs 33.8m, Log rank test, χ2 = 1.048, P=0.305). (G). mOS was not significantly different between low TMB and high TMB patients (NR vs 33.8m, Log rank test, χ2 = 0.104, P=0.747). [file DataSheet_3.pdf]

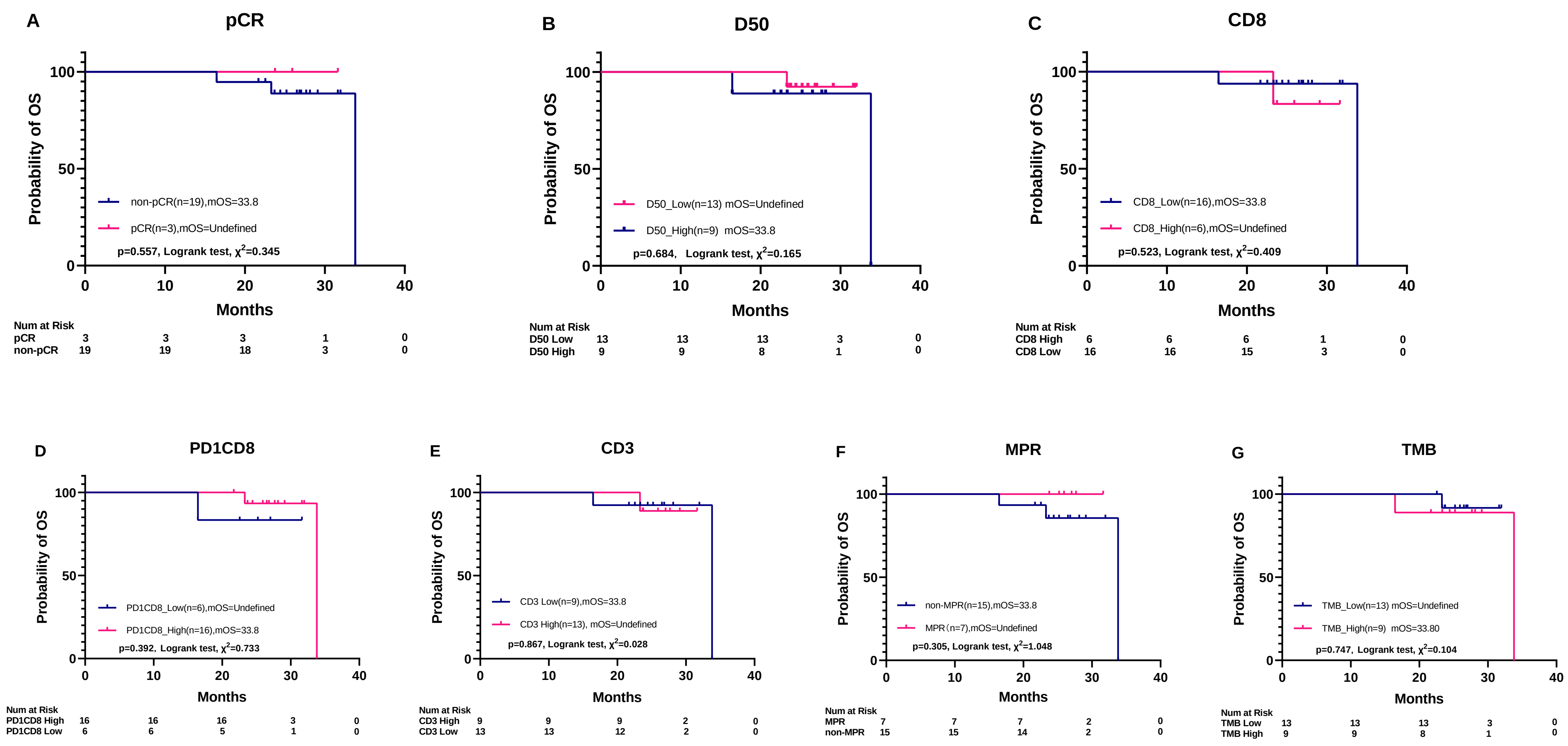
A
pCR
Probability of OS
100
50
0
0
10
20
30
40
Months
non-pCR(n=19),mOS=33.8
pCR(n=3),mOS=Undefined
p=0.557, Logrank test, χ²=0.345
Num at Risk
pCR 3 3 3 1 0
non-pCR 19 19 18 3 0
B
D50
Probability of OS
D50_Low(n=13) mOS=Undefined
D50_High(n=9) mOS=33.8
p=0.684, Logrank test, χ²=0.165
Months
Num at Risk
D50 Low 13 13 13 3 0
D50 High 9 9 8 1 0
C
CD8
Probability of OS
CD8_Low(n=16),mOS=33.8
CD8_High(n=6),mOS=Undefined
p=0.523, Logrank test, χ²=0.409
Months
Num at Risk
CD8 High 6 6 6 1 0
CD8 Low 16 16 15 3 0
D
PD1CD8
Probability of OS
PD1CD8_Low(n=6),mOS=Undefined
PD1CD8_High(n=16),mOS=33.8
p=0.392, Logrank test, χ²=0.733
Months
Num at Risk
PD1CD8 High 16 16 16 3 0
PD1CD8 Low 6 6 5 1 0
E
CD3
Probability of OS
CD3 Low(n=9),mOS=33.8
CD3 High(n=13), mOS=Undefined
p=0.867, Logrank test, χ²=0.028
Months
Num at Risk
CD3 High 9 9 9 2 0
CD3 Low 13 13 12 2 0
F
MPR
Probability of OS
non-MPR(n=15),mOS=33.8
MPR（n=7),mOS=Undefined
p=0.305, Logrank test, χ²=1.048
Months
Num at Risk
MPR 7 7 7 2 0
non-MPR 15 15 14 2 0
G
TMB
Probability of OS
TMB_Low(n=13) mOS=Undefined
TMB_High(n=9) mOS=33.80
p=0.747, Logrank test, χ²=0.104
Months
Num at Risk
TMB Low 13 13 13 3 0
TMB High 9 9 8 1 0
